# Prediction and Characterization of High-Activity Events in Social Media Triggered by Real-World News

**DOI:** 10.1371/journal.pone.0166694

**Published:** 2016-12-16

**Authors:** Janani Kalyanam, Mauricio Quezada, Barbara Poblete, Gert Lanckriet

**Affiliations:** 1 Department of Electrical and Computer Engineering, University of California San Diego, La Jolla, California, United States of America; 2 Department of Computer Science, University of Chile, Santiago, Chile; Universite de Namur, BELGIUM

## Abstract

On-line social networks publish information on a high volume of real-world events almost instantly, becoming a primary source for breaking news. Some of these real-world events can end up having a very strong impact on on-line social networks. The effect of such events can be analyzed from several perspectives, one of them being the intensity and characteristics of the collective activity that it produces in the social platform. We research 5,234 real-world news events encompassing 43 million messages discussed on the Twitter microblogging service for approximately 1 year. We show empirically that exogenous news events naturally create collective patterns of bursty behavior in combination with long periods of inactivity in the network. This type of behavior agrees with other patterns previously observed in other types of natural collective phenomena, as well as in individual human communications. In addition, we propose a methodology to classify news events according to the different levels of intensity in activity that they produce. In particular, we analyze the most highly active events and observe a consistent and strikingly different collective reaction from users when they are exposed to such events. This reaction is independent of an event’s reach and scope. We further observe that extremely high-activity events have characteristics that are quite distinguishable at the beginning stages of their outbreak. This allows us to predict with high precision, the top 8% of events that will have the most impact in the social network by just using the first 5% of the information of an event’s lifetime evolution. This strongly implies that high-activity events are naturally prioritized collectively by the social network, engaging users early on, way before they are brought to the mainstream audience.

## Introduction

Social media is now a primary source of breaking news information for millions of users all over the world [1]. On-line social networks along with mobile internet devices have crowdsourced the task of disseminating real-time information. As a result, both news media and news consumers have become inundated with much more information than they can process. One possible way of handling this data overload, is to find ways to filter and prioritize information that has the potential of creating a strong collective impact. Understanding and quickly identifying the type of reaction that certain exogenous events will produce in on-line social networks, at both global and local scales, can help in the understanding of collective human behavior, as well as improve information delivery, journalistic coverage and crisis management, among other things. We address this challenge by analyzing the properties of real-world news events in on-line social networks, showing that they corroborate patterns previously identified in other case studies of human communications. In addition, we present our main findings of how news events that produce extremely high-activity can be clearly identified in the early stages of their outbreak.

The study of information propagation on the Web has sparked tremendous interest in recent years. Current literature on the subject primarily considers the process through which a *meme*, usually a piece of media (like a video, an image, or a specific Web article), gains popularity [2–9]. However, a meme represents a simple information unit and its propagation behavior does not necessarily correspond to that of more complex information such as news events. News events are usually diffused in the network in many different formats, e.g., a particular news story such as an *earthquake in Japan* can be communicated through images, URLs, tweets, videos, etc. Therefore, current research can benefit from analyzing the effects of more high-level forms of information.

**Fig 1:**
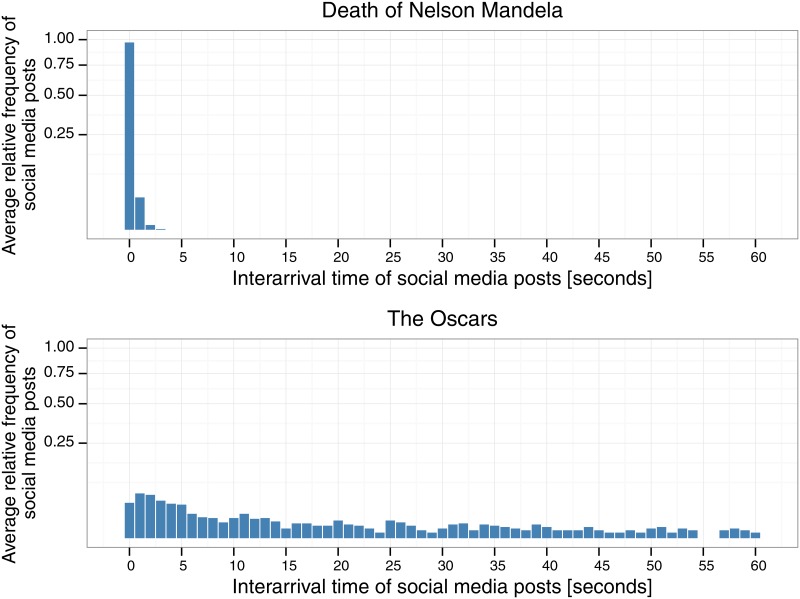
Examples of interarrival time histograms of two real-world news events discussed on Twitter. The event [nelson, mandela] (top) was collected on 12/05/2013. Since there is a high concentration in the first histogram bin, we conclude that most of the social media posts for this event occur in one or more successions of high-activity bursts (therefore, considered a high-activity event). The second event, [may, oscar] (bottom) was collected on 03/23/2014 about The Oscars event that was held a few weeks before. The arrival times of these posts are much more spread out, displaying much less concentration of bursty activity.

Traditionally, the impact of information in on-line social networks has been measured in relation to the total amount of attention that this subject receives [10–14]. That is, if a content posted in the network receives votes/comments/shares above a certain threshold it is usually deemed as *viral* or *popular*. Nevertheless, this notion of popularity or impact will favor only information that produces very large volumes of social media messages. Naturally, global breaking news that has world-wide coverage and that produces a high volume of activity in a short time should be considered as having a strong impact on the network. However, there are other types of events that can produce a similar reaction in smaller on-line communities such as, for example, on users from a particular country (e.g., the withdrawal of the main right wing presidential candidate in Chile due to psychiatric problems, just before elections [15]). Clearly, events of local scope do not produce as much social media activity as events of global scope, but they can create a strong and immediate reaction from users in local networks [16]. Conversely, there are large events which do not produce an intense reaction, such as *The Oscars* (Fig 1), which span a long period of time and are discussed by social network users for weeks or even months, but do not spark intense user activity. Therefore, it is reasonable to consider additional dimensions, than just volume, when analyzing the impact of information in on-line communities.

Prior research has shown that certain types of individual activities, such as communications (studied in email exchanges), work patterns and entertainment, follow a behavior of bursts of rapidly occurring actions followed by long periods of inactivity [17], referred to as temporally inhomogeneous behavior [18]. This type of behavior initially observed in individual activities, has also been observed in relation to other naturally occurring types of collective phenomena in human dynamics similar to processes seen in self-organized criticality [18]. In particular, extremely high-activity bursty behavior seems to also occur in critical situations, observed from the information flow in cell phone networks during emergencies [19]. Although, there is research towards modeling this type of collective behavior [20] in on-line social networks, to the best of our knowledge, it has not yet been analyzed quantitatively.

Our work focuses on high-activity events in social media produced by real-world news, with the following contributions:

1. We introduce a methodology for modeling and classifying events in social media, based on the intensity of the activity that they produce. This methodology is independent of the size and scope of the event, and is an indicator of the impact that the event information had on the social network.
2. We show empirically that real-world news events produce collective patterns of bursty behavior in the social network, in combination with long periods of inactivity. Furthermore, we identify events for which most of their activity is concentrated into very high-activity periods, we call these events *high-activity events*.
3. We determine the existence of unique characteristics that differentiate how high-activity events propagate in the social network.
4. We show that an important portion of high-activity events can be predicted very early in their lifecycle, indicating that this type of information is spontaneously identified and filtered collectively, early on, by social network users.

## Materials and Methods

We define an event as a conglomerate of information that encompasses all of the social media content related to a real-world news occurrence. Using this specification, which considers an event as a complex unit of information, we study the type of collective reaction produced by the event on the social network. In particular, we analyze the intensity or immediacy of the social network’s response. By analyzing the levels of intensity in activity induced by different exogenous events to the network, we are implicitly studying the priority that has been collectively assigned to the event by groups of independent individuals [17, 18].

We characterize an event’s discrete activity dynamics by using *interarrival times* between consecutive social media messages within an event (e.g., *d*_*i*_ = *t*_*i*+1_ − *t*_*i*_, where *d*_*i*_ denotes the interarrival time between two consecutive social media messages *i* and *i* + 1 that arrived in moments *t*_*i*_ and *t*_*i*+1_, respectively).

We introduce a novel vectorial representation based on a *vector quantization of the interarrival time distribution*, which we call *“VQ-event model”*. This model is designed to filter events based on the distribution of the interarrival times between consecutive messages. This approach is inspired by the *codebook-based representation* from the field of multimedia content analysis, which has been used in audio processing and computer vision [21, 22]. In our proposed approach, our method learns a set of the most representative interarrival times from a large training corpus of events; each one of the representative interarrival times is known as a *codeword* and the complete learned set is known as the *codebook* [22]. Each event is then modeled using a vector quantization (VQ) that converts the interarrival times of an event into a discrete set of values, each value corresponding to the closest codeword in the codebook (details in supplementary material). The resulting VQ-event model is then a vector in which each dimension contains the percentage of interarrival times of the event that were assigned a particular codeword in the codebook.

The VQ-event representation is relative to an event’s overall size since the model is normalized with respect to the number of messages in the event. Therefore the only criteria that are considered in the model are the interarrival times of each particular event. This model allows us to group events based on the *similarity of the distribution* of their interarrival times. In those terms, we consider as high-activity events those events for which the distribution of interarrival times is most heavily skewed towards the smallest possible interval, zero. In other words, events for which the overall activity is extremely intense in comparison with other events.

To illustrate events with different levels of intensity in activity we present two examples taken from our analysis of Twitter data. These examples show the interarrival time histograms for the entire lifecycle of the two events. In the first example, the majority of the messages about the death of political leader Nelson Mandela (Fig 1) arrive within almost zero seconds of each other. On the contrary, the messages about The Oscars (Fig 1) are much more spread out in time.

We note that, by using interarrival times to describe the intensity of the activity of an event, we make our analysis independent of the particular evolution of each event. By doing this, we put no restrictions on how high-activity events unfold in time, for example, they could be: (a) events that start out slowly and suddenly gain momentum, (b) events that go viral soon after they appear on social media and then decay in intensity over a long (or short) period of time, (c) events that from the beginning produce large amounts of interest and sustain that interest throughout their long (or short) lifespan, or (d) events that are a concatenation of any of the above, etc.

We study a dataset of news events gathered from news headlines from a *manually curated* list of well-known news media accounts (e.g., @CNN, @BreakingNews, @BBCNews, etc.) in the microblogging platform Twitter [23] (a full list of all the news media accounts is provided in the supplementary material). Headlines were collected periodically every hour, over the course of approximately one year. In parallel, all the Twitter messages (called *tweets*) were extracted for each news event using the public API [24]. This process was performed by automatically extracting descriptive sets of keywords for each event using a variation of frequent itemset extraction [25] over the event’s headlines. These sets of keywords were then used to retrieve corresponding user tweets for each event. We validate the events gathered in our data collection process to ensure that each group of social media posts corresponds to a meaningful and cohesive news event. We provide a detailed description of the collection methodology and of the validation of event cohesiveness in the supplementary material. Overall, the resulting dataset contains 43,256,261 tweets that account for 5,234 events (Table 1).

**Table 1:** High-level description of the dataset of news events.

Event Collection Statistics	Minimum	Mean	Median	Maximum
# of posts (per event)	1,000	8,254	2,474	510,920
# of keywords (per tweet)	2	3.77	3	39
Event duration (hours)	0.12	20.93	7.46	190.43

**Fig 2:**
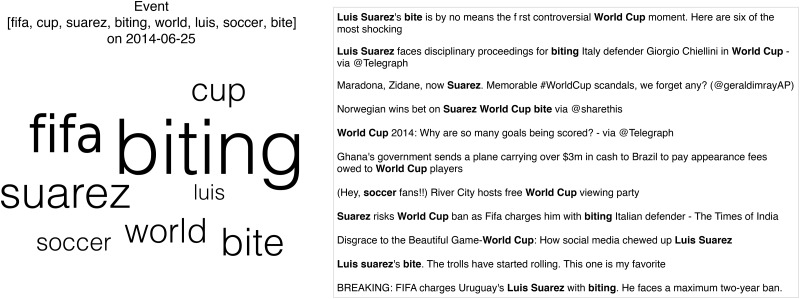
An example event, collected on 06/25/2014 with keywords (left) and sample user posts (right) obtained from the Twitter Search API. The tweets in the event contain at least a pair of descriptive keywords and were retrieved close to the time of the event.

In Fig 2 we characterize an example event from our dataset, by showing the set of keywords and a sample of tweets associated to the event. These keywords form a semantically meaningful event; they refer to the incident where soccer player Luis Suarez was charged for biting another player during the FIFA World Cup in 2014. This general collection process results in a set of social media posts associated to an event which can encompass several memes, viral tweets and pieces of information. Therefore, an event is composed of diverse information, addressing more heterogeneous content than prior work [2–4, 6, 7, 26, 27] which focus on single pieces of information (e.g., a particular meme, a viral tweet etc.).

**Fig 3:**
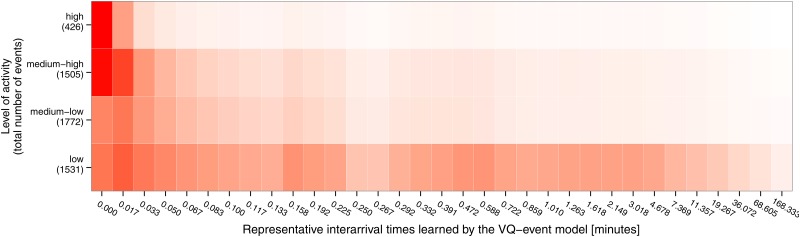
Each row is the average representation of all the events in a cluster. A darker cell represents a higher relative frequency value. The y-axis specifies the number of events in each cluster. Clusters are (top to bottom): high-activity, medium-high medium-low and low.

The collection of events is converted into their VQ-event model representation. Using this model, we can identify events that have produced similar levels of activity in the social network. In other words, events are considered to have similar activity if the interarrival times between their social media posts are similarly distributed, implying a very much alike collective reaction from users to the events within a group. In order to identify groups of similar events, we cluster the event models. We sort the resulting groups of events from highest to lowest activity, according to the concentration of social media posts in the bins that correspond to short interarrival times. We consider the events that fall in the top cluster to be high-activity events as most of their interarrival times are concentrated in the smallest interval of the VQ-event model. In our dataset, these correspond to roughly 8% of the events. We consider the next clusters in the sorted ranking to form medium-high activity events, and so on. Thus we end with four groups of events: high, medium-high, medium-low and low. Fig 3 shows a heatmap of the interarrival relative frequency for each cluster. This classification of events based on activity intensity is independent of event size. More details of this methodology are provided in S1 Appendix.

## Results and Discussion

Our main objective in this work is to analyze the characteristics of high-activity events which differentiate them from other types of events. In particular, we identify how early on in an event’s lifecycle can we determine if an event is going produce high activity in the on-line social network.

Tables 2 and 3 show examples of events from the high-activity category and low-activity category. We recall that the high-activity events are those which were in the top 8% of the ranking obtained by sorting the event clusters according to concentration of interarrival times of social media posts in the shortest interarrival time of the VQ-event model. Table 2 shows two events of different sizes (large and small) and different scopes (one global and the other of more local scope) categorized as high activity in our dataset. The first event, the death of Nelson Mandela, is one of the largest events in the dataset, with ≈ 134,000 tweets. The histogram representation of this event, shown in Fig 1, suggests that more than 80% of the activity of the event was produced in high-activity periods. This is an event of international, political, and social importance, that produced an overwhelming flood of messages on social media. Hence, it makes sense for such an example to be a high-activity event. The second event, on the other hand, about the 2013 Mumbai Gang Rape is of much smaller scale, with a total of ≈ 1,700 tweets. However, this event caused considerable amount of immediate reaction on social media, with close to 50% of its activity concentrated within high-activity periods. Despite its smaller size, in comparison to the previous event, this event displays a similar reaction to that of other high-activity events, but at a smaller scale.

**Table 2:** Examples of high-activity news events. The events shown were taken from the “high” category according to Fig 4.

Event	Sample Tweets
**Description:** Death of South African politician Nelson Mandela.	@DaniellePeazer: RIP Nelson Mandela….. what a truly phenomenal and inspirational man xx
**Keywords:** [nelson, mandela]	@iansomerhalder: Im in tears. The world has lost one of its greatest shepherds of peace. Thank you Mr.Mandela for the love you radiated. http://t.co/u39MVVEKe8
**Date:** 2013-12-05	@FootballFunnys: This is so true. RIP Nelson Mandela. http://t.co/vF9xri8LdP
**Size:** 134,637 tweets	@David_Cameron: I’ve spoken to the Speaker and there will be statements and tributes to Nelson Mandela in the House on Monday.
**Description:** 2013 Mumbai Gang Rape	@TheNewsRoundup: Mumbai gang-rape: Second accused confesses to crime: Mumbai Police—Daily News Analysis http://t.co/KnabwhqH66
**Keywords:** [rape, mumbai]	@vijayarumugam: An interesting take on the Mumbai rape: http://t.co/ylBmW4l8sA
**Date:** 2013-08-24	@LondonStephanie: Two arrested over gang rape of Mumbai photojournalist that sparked renewed protests in India http://t.co/McYfLNDvaE
**Size:** 1,705 tweets	@GanapathyI: Most brutal rapist of Delhi gang-rape was 17. Most brutal rapist of Mumbai gang-rape is 18. Worst Young generation I have seen in my life.

**Table 3:** Examples of events with low activity. The events shown were taken from the “low” category according to Fig 4.

Event	Sample Tweets
**Description:** Teen survives hiding in a plane wheel.	@ToniWoemmel: 16-year-old somehow survives flight from California to Hawaii stowed away in planes wheel well: http://t.co/IGiJa60SiK
**Keywords:** [teen, survives, old, well, skydivers, plane, wheel, flight]	@iOver_think: 38,000 feet at -80F: Teen stowaway survives five-hour California-to-Hawaii flight in wheel well http://t.co/ejXQH9VZyT
**Date:** 2014-04-21	@TruEntModels: GOD IS GOOD…runaway TEEN hid in plane’s wheel for 5 HOUR flight during FREEZING temps and survived http://t.co/6g6Cqhs9Ib
**Size:** 18,519	@DvdVill: A 16-year-old kid, who was mad at his parents, hid inside a jet wheel and survived flight to Hawaii. http://t.co/c82GbjrfUH
**Description:** Surveying the damages of recent tornado in Canada.	@Kathleen_Wynne: Visited #Angus today to survey the damage. Thankfully no fatalities or major injuries from recent tornado. http://t.co/xRQyRWg5Vw
**Keywords:** [canada, tornado]	@SunNewsNetwork: PHOTOS & VIDEO: Hundreds displaced after tornado hits Ontario town, destroying homes http://t.co/L38rG6N1a6
**Date:** 2014-06-21	@CBCToronto: Kathleen Wynne is speaking at site of tornado damage in Angus, Ont. now. Watch live here: http://t.co/EDKNUiZo0X #cbcto
**Size:** 1,033	@InsuranceBureau: @CTVBarrieNews: Insurance Bureau of Canada is setting up a mobile unit in #Angus today to help residents affected by #Tornado

Table 3 shows events that have been classified by our methodology in the category of low activity. The first event, about a teen surviving after hiding in the wheel of a airplane, had only a little more than 25% of its messages arriving with high-activity bursts although it had over 18,000 messages. The second event, about the damages caused by a tornado in Canada, did not garner much immediacy in attention of Twitter users, with only 7% of its messages produced with short interarrival times. Most of the messages of this event were well spaced out in time. Even though we cannot say whether or not this event had significant implications in the real-world, we can say that it did not have considerable impact on the Twitter network. The lack of interest could be due to several factors that are currently beyond the scope of this work, ranging from the lack of Twitter users in the locality of the real-world event, to it not being considered urgent by Twitter users. We intend to research the relation between the real-world impact of an event and the network reaction in future work.

**Fig 4:**
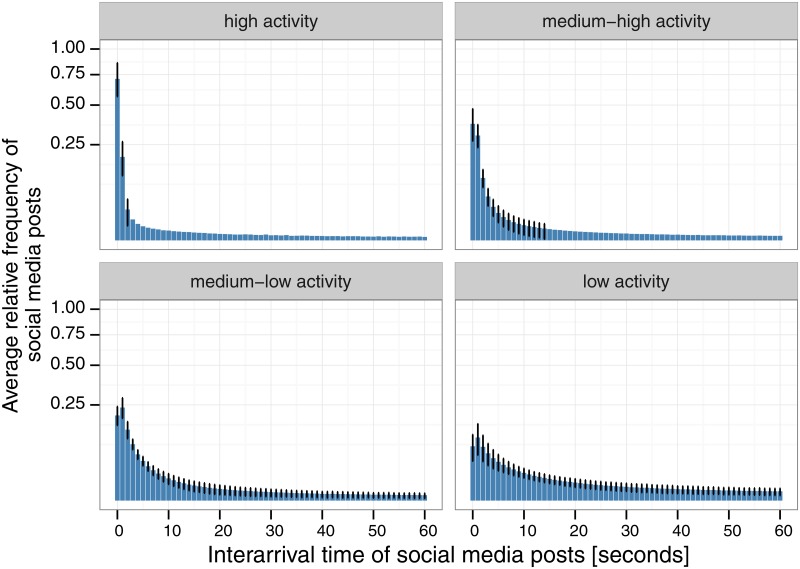
Average histograms of the high activity, medium-high activity, medium-low activity and low activity clusters in our dataset (from left to right and top to bottom). All histograms include standard deviation bars and were cut-off at 60 second length for better visibility.

**Fig 5:**
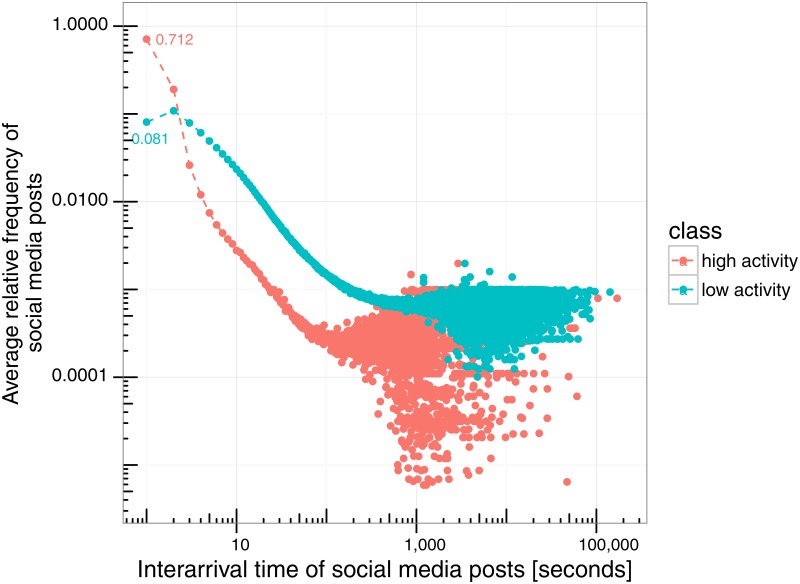
Scatter plots of the average relative frequencies of interarrival times for the high-activity and low-activity clusters of events (i.e., scatter plots of the histograms in Fig 4 in log-log scale). *y*-axis represents the average relative frequency of social media messages and *x*-axis the interarrival time.

**Fig 6:**
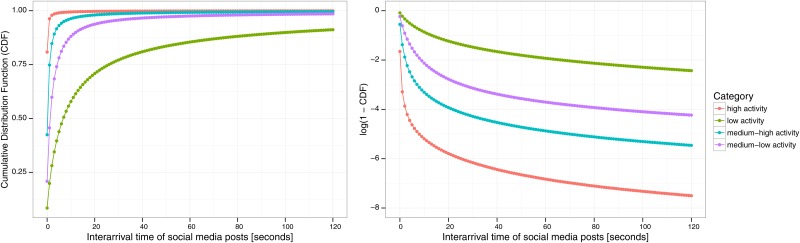
(Left) Average cumulative distribution function (CDF) for the high activity, medium-high activity, medium-low activity and low activity clusters in our dataset. (Right) log(1−CDF) for the same clusters.

Fig 4 shows the average histograms for events that belong to the high activity, medium-high activity, medium-low activity and low-activity clusters (displayed from left to right and top to bottom). All histograms show a quick decay in average relative frequency (resembling a distribution from the exponential family). In particular, the high-activity group concentrates most of its activity in the shortest interarrival rate, with lower activity groups mostly concentrating their activity in the second bin with slower decay. Fig 5 further characterizes the differences in behavior of the high and low-activity groups, showing that high-activity events concentrate on average 70% fo their activity in the smallest bin (0 sec.), against 8% for low-activity events. In addition, Fig 6 (left) shows the cumulative distribution function (CDF) for each group of events, and Fig 6 (right) shows log(1−CDF). Visual inspection shows a clear difference in how interarrival rates are distributed within each group, however, these figures do not indicate a power-law distribution nor exponential distribution.

Further analysis of the high-activity events shows significant differences to other events, in the following aspects: (i) how the information about these events is propagated, (ii) the characteristics of the conversations that they generate, and (iii) how focused users are on the news topic. In detail, high-activity events have a higher fraction of *retweets* (or shares) relative to their overall message volume. On average, a tweet from a high-activity event is retweeted 2.36 times more than a tweet from a low activity event. The most retweeted message in high-activity events is retweeted 7 times more than the most retweeted message in a medium or low activity event. We find that a small set of initial social media posts are propagated quickly and extensively through the network without any rephrasing by the user (just plain forwarding). Intuitively, this seems justified given general topic urgency of high-activity events. Events that are not high-activity did not exhibit these characteristics.

Our research also revealed that high-activity events tend to spark more conversation between users, 33.4% more than other events. This is reflected in the number of *replies* to social media posts. The number of different users that engage with high-activity events is 32.7% higher than in events that are not high-activity. Posts about high-activity events are much more topic focused than in other events. The vocabulary of unique words as well as *hashtags* used in high-activity events is much more narrow than for other events. Medium and low activity events have over 7 times more unique hashtags than high-activity events. This is intuitive, given that if a news item is sensational, people will seldom deviate from the main conversation topic.

In a real-world scenario, in order to predict if an early breaking news story will have a considerable impact in the social network, we will not have enough data to create its activity-based model, i.e., we will not yet know the distribution of the speed at which the social media posts will arrive for the event. For instance, an event can start slowly and later produce an explosive reaction, or start explosively and decay quickly to an overall slower message arrival rate. Still, reliable early prediction of very high-activity news is important in many aspects, from decisions of mass media information coverage, to natural disaster management, brand and political image monitoring, and so on.

For the task of early prediction of high-activity events we use features that are independent of our activity-based model such as the retweets, the sentiment of the posts about the event, etc. These features are computed on the early 5% of messages about the event. The results are an average from a 5-fold cross validation with randomly selected 60% training, 20% validation and 20% test splits. The high-activity events are identified with a precision of 82% using only the earliest 5% of the data of each event (Table 4). Additionally, we were able to identify with high accuracy a considerable percentage of all high-activity events (≈ 46%) at an early stage, with very few false positives (Tables 4 and 5).

**Table 4:** Classification of high-activity events.

	Early 5% Tweets				All Tweets			
	FP-Rate	Precision	Recall	ROC-area	FP-Rate	Precision	Recall	ROC-area
high-activity	0.009	0.819	0.455	0.900	0.01	0.830	0.540	0.945
non-high-activity	0.545	0.954	0.991	0.900	0.460	0.960	0.990	0.945

**Table 5:** Confusion matrix for high-activity events prediction.

	Early 5% Tweets 2c		All Tweets	
	high-activity	non-high-activity	high-activity	non-high-activity
high-activity	194	232	230	196
non-high-activity	43	4,765	47	4,761

The precision using only the early tweets is almost as good as using all tweets in the event (0.819 to 0.830). This suggests that the social network somehow acts as a natural filter in separating out the high-activity events fairly early on. The recall goes from 0.455 to 0.540. This indicates that there are some high-activity events which require more data in order to determine what kind of activity they will produce, or events for which activity occurs due to random conditions. A detailed description of the features and different classification settings are provided in the supplementary material.

## Conclusion

We study the characteristics of the activity that real-world news produces in the Twitter social network. In particular, we propose to measure the impact of the real-world news event on the on-line social network by modeling the user activity related to the event using the distribution of their interarrival times between consecutive messages. In our research we observe that the activity triggered by real-world news events follows a similar pattern to that observed in other types of collective reactions to events. This is, by displaying periods of intense activity as well as long periods of inactivity. We further extend this analysis by identifying groups of events that produce much more concentration of high-activity than other events. We show that there are several specific properties that distinguish how high-activity events evolve in Twitter, when comparing them to other events. We design a model for events, based on the codebook approach, that allows us to do unambiguous classification of high-activity events based on the impact displayed by social network. Some notable characteristics of high-activity events are that they are forwarded more often by users, and generate a greater amount of conversation than other events. Social media posts from high-activity news events are much more focused on the news topic. Our experiments show that there are several properties that can suggest early on if an event will have high-activity on the on-line community. We can predict a high number of high-activity events *before* the network has shown any type of explosive reaction to them. This suggests that users are collectively quick at deciding whether an event should receive priority or not. However, there does exist a fraction of events which will create high activity, despite not presenting patterns of other high activity events during their early stages. These events are likely to be affected by other factors, such as random conditions found in the social network at the moment and require further investigation.

## Supporting Information

S1 Appendix (PDF)
