# Supplementary material for: Prediction and Characterization of High-Activity Events in Social Media Triggered by Real-World News
Source: PLoS One. 2016 Dec 16;11(12):e0166694. doi: 10.1371/journal.pone.0166694 (PMC5161319; doi:10.1371/journal.pone.0166694)
Supplement: S1 Appendix — (PDF) [file pone.0166694.s001.pdf]

1 Data Collection Methodology

The Twitter Search API[1] was used to obtained tweets about $5,234$ news events. This encompasses a total of $43,256,261$ tweets. Table 1 shows a high level description of the dataset. The full dataset is available in `http://dcc.uchile.cl/~mquezada/breakingnews/`.

News events' property	Minimum	Mean	Median	Maximum
# of tweets	$1,000$	$8,254$	$2,474$	$510,920$
# of keywords	2	3.77	3	39
Event duration (hours)	0.12	20.93	7.46	190.43

Table 1: **High-level description of the dataset of news events.**

1.1 Collecting the Tweets

The data collection process entails detecting pairs of keywords from the most recent hourly batch of news headlines (the pairs of keywords are meant to describe the events succinctly), and then searching for tweets using the pairs of keywords as queries. We merge the search results of 'similar' queries every 24 hours and form the tweets set for an event. We obtained the hourly batch of headlines from the news media accounts on Twitter. The news accounts were obtained by performing a search for accounts in the twitter page using 'news' as keyword, and then manually selecting the verified accounts resulting from the search result. Figure 1 represents the high-level flowchart of the data collection process. A summary of this process is described in Algorithm 1. The accounts are verified accounts on Twitter[2].

In Algorithm 1, the goal of the `detect_keywords()` module is to produce pairs of keywords that coherently, and succinctly describes an event. Inspired by the data mining concept of mining frequent itemsets (*2*), we develop an algorithm which identifies the most commonly occurring keyword groups (or item sets) in the headlines. From the item sets, we pick the most common keyword pairs. The algorithm is described in Algorithm 2. This algorithm finds string intersections between headlines (`intersect()` in Line 5 returns the number of words present in both s_a and s_b). If the common set of words has sufficient Jaccard similarity to any of the existing item sets, then the common set of words are added to that item set. If not, a new item set is created (Line 11). During the process of identifying the most commonly occurring item sets, we also track how many times each keyword has been added to an item set, namely, the score of the keyword. The score of each item set is the average of the scores of its keywords. Once the item sets have been identified, we select the top 2 keywords from each of the top six

[1]`http://dev.twitter.com` (Accessed: August 25, 2015)

[2]Verified accounts on Twitter establish authenticity of identity of key individuals and organizations.

item sets and use them for searches. We preprocess the headlines to remove duplicates, stop-words, punctuation, convert everything to lower case, and subject the text through the process of stemming.

We made the choice of selecting 2 keywords since having a single keyword maybe not define an event accurately. For example, the keyword {obama} could retrieve tweets about any event related to Obama. However, a keyword *pair* like {obama, syria} describes the event more accurately[3].

The Twitter Search API imposes several restrictions on the number of searches that can be performed in a given time duration. We produce six search threads to perform searches, one for each keyword pair. All in all, with $\tau = 60$ minutes in Figure 1, six new pairs of keywords are discovered from the most recent batch of headlines, and then we query for tweets in the Twitter Search API using these keywords over the next one hour.

We make some notes about the data collection methodology. Firstly, there is a temporal sensitivity to the data collection methodology. For example, one of the keyword pairs obtained as soon the Malaysian airlines jet disappeared was {plane,missing}. Although this keyword pair does not specifically refer to the Malaysian airlines jet, it is likely that the tweets retrieved from searching for this pair will indeed be about the Malaysian airlines plane that went missing, since the search is performed as and when the event breaks out. Secondly, Algorithm 2 may return multiple pairs of keywords (possibly different pairs) describing the same event. Some pair examples of keywords produced when there was a bomb threat at Harvard University in December 2013 were {harvard, evacuated}, {harvard, explosives}, etc. How do we merge the keyword pairs which belong to the same event? In order to address this, we collect all the pairs obtained in the past 24 hours, and build a graph with keywords as nodes, and keyword pairs (as obtained from Algorithm 2) as edges. We then discover the connected components of this graph, and treat each connected component as an "event"[4]. The set of tweets obtained by merging the tweets from each of the keyword pairs is the set of messages associated with the event. Figure 4 is an example component formed on December 16, 2013. It illustrates the merge of smaller keyword pairs into larger components for two events. One was the bomb threat at Harvard University, and the other was about the attack on police in the Xinjiang province in China.

1.2 Cleaning the Data

The data was preprocessed to reduce the noisy and irrelevant tweets.

[3]Having more than two keywords may impose too much of a restriction on the query, leading to little or no tweets in the retrieval.

[4]For the rest of the document, the terms *connected component* and *event* are used interchangeably. Both of them refer to the definition of *event* given in the main article.

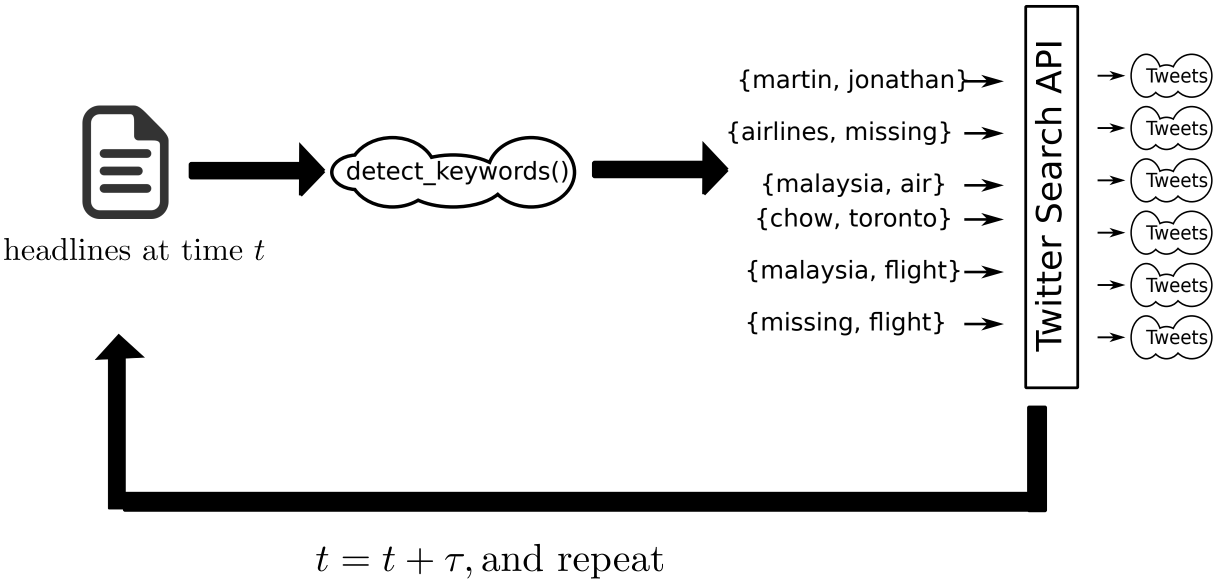

Figure 1: **This figure illustrates the high level data collection process. Headlines are collected every hour, and 6 keyword pairs are chosen to search for tweets. These keyword pairs are detected with the goal of concisely representing queries for an event.**

Algorithm 1 `data_collection()`

Input: stream of headlines.
Output: data structures $\{\mathcal{H}_1, \mathcal{H}_2, \ldots\}$, with $\mathcal{H}.keywords$ = keyword pair, and $\mathcal{H}.tweets$ = set of tweets

$i \leftarrow 0$, $j \leftarrow 0$
loop
  $\mathcal{S} \leftarrow$ headlines for hour-i
  $keyPairs \leftarrow$ `detect_keywords`$(\mathcal{S})$ $\{keyPairs$ is a list of keyword pairs.$\}$
  for $k = 0$ to `len`$(keyPairs)$ -1 **do**
    $\mathcal{H}_j.keywords \leftarrow keyPairs[k]$
    $\mathcal{H}_j.tweets \leftarrow$search$(\mathcal{H}_j.keywords)$ {using Twitter Search API}
    $j \leftarrow j + 1$
  end for
  $i \leftarrow i + 1$
end loop

Algorithm 2 `detect_keywords()`

Input: A set of M sets of words, $\mathcal{S} = \{H_1, H_2, \ldots, H_M\}$, positive integers k, η
Output: k sets of keywords, $G = (\mathcal{I}_1, \mathcal{I}_2, \ldots, \mathcal{I}_k)$

$\mathcal{I}_i \leftarrow \emptyset$ for $i = 1, 2, \ldots, k$
$score_i \leftarrow$ empty dictionary for $i = 1, 2, \ldots, k$
$i \leftarrow 1$
for every pair of headlines $\{H_a, H_b\} \in \mathcal{S}$ such that $|H_a \cap H_b| \geq \eta$ **do**
    $\mathcal{G} \leftarrow H_a \cap H_b$
    $j \leftarrow \arg\max_j |\mathcal{I}_j \cap \mathcal{G}|$
    if $|\mathcal{I}_j \cap \mathcal{G}| \geq \eta$ **then**
        $\mathcal{I}_j \leftarrow \mathcal{I}_j \cap \mathcal{G}$
        $score_j[w] \leftarrow score_j[w] + 1$ for all $w \in \mathcal{I}_j$
    else
        $\mathcal{I}_i \leftarrow \mathcal{G}$
        $score_i[w] \leftarrow 1$ for all $w \in \mathcal{I}_i$
        $i \leftarrow i + 1$
    end if
end for
$total\_score_i \leftarrow \sum_{w \in \mathcal{I}_i} score_i[w]$ for $i = 1, 2, \ldots, k$
return $G \leftarrow$ ($\mathcal{I}_i$ sorted by $total\_score_i$)

1.2.1 Special Stopwords: Articulation Words

During the data collection process, sometimes unrelated events were joined together with keywords that was common to both events.

Typical stopwords such as "the" and "a" were removed during preprocessing the news headlines. However, there are other words which occur quite commonly in news headlines. For example, words like "watch", "live", or "update" are common to express things like "watch this video", "we are live on TV", or to update a previous headline with more information. Such words could possibly incorrectly connect two or more very different events as one. Example: "Watch Jim Harbaugh's press conference live"[5] and "WATCH LIVE: Of the 48 people being monitored for contact with Dallas patient, no one is showing any symptoms"[6]. We call such words *articulation words* We now delve into understanding how and when these words occur, and how to subsequently identify and remove them in the preprocessing step, just as we would a stopword.

It is well known that *tf-idf* (*1*) is a statistic of a word that indicates how important that word is in a given document. Intuitively, if a word appears in all the documents, then its statistic is generally low in all the documents. However, if the word appears in very few documents, its

[5] `https://twitter.com/49ers/status/519202023628374016` (Accessed: August 25, 2015)
[6] `https://twitter.com/PzFeed/status/519203692898435072` (Accessed: August 25, 2015)

statistic in those documents is fairly high, indicating that the word is somehow representative of the content of the document. It turns out the articulation words do not occur often enough for them to be detected by regular *tf-idf*, but do occur enough times for them to falsely relate several unrelated events together. To identify a group of those keywords, we used a modified *tf-idf* to detect them from the headlines.

The modified version of *tf-idf*, what we refer as *maxtf-idf*, is meant to assign more weight to the terms that are frequent in any document. For instance, *tf-idf* of a term in a document tries to assign a weight related to how "rare" that term is in the whole collection, and how frequent the term is in that document, thus indicating how representative the term is of the document. On the other hand, we want to place a higher weight on a term if its frequency is higher in any other document, relative to the frequency in the current document. With that in mind, we want to identify terms that might be "adding noise" to the corpus and hence merge unrelated events together.

The definition of *maxtf* is as follows:

$$\mathrm{maxtf}(t, d, D) = 0.5 + \frac{0.5 + max\{f(t, d') : d' \in D\}}{max\{f(w, d) : w \in d\}} \tag{1}$$

and for *idf*, the usual formula:

$$\mathrm{idf}(t, D) = \log \frac{N}{|\{d \in D : t \in d\}|} \tag{2}$$

where t is a term, d is a document, and D is the corpus of all documents. In this case, we set t as a keyword, d as the set of keywords of one hour of a given day, and D the set of documents of that day.

After identifying such words, the idea is to disconnect the components connected by those words. The process is to disconnect each component by the word with top normalized $1 -$ maxtf-idf score each time until the component could not be disconnected further. We add the top scoring words to our list of stopwords. These words are hence ignored from the subsequent runs of the data collection methodology. In Figure 2 there are two examples of this process to identify the words.

1.2.2 Discarding Irrelevant Tweets

Due to the capabilities of the REST API, the tweets collected can be older than the actual date of the event detected. Hence, some tweets can be very old and not relevant to the event itself. This may lead to inaccuracies in predictions when using the early features.

This problem is illustrated in Figure 3, Note that the first 5% of the tweets take an unusually large portion of the duration of the entire event. This suggests that we are collecting tweets which existed much before the event broke out, and hence are possibly irrelevant. Once we discard the first 5% of tweets, we observe that each segment of the event (first 5%, the next 5%, etc.) occupies roughly the same duration of the entire event.

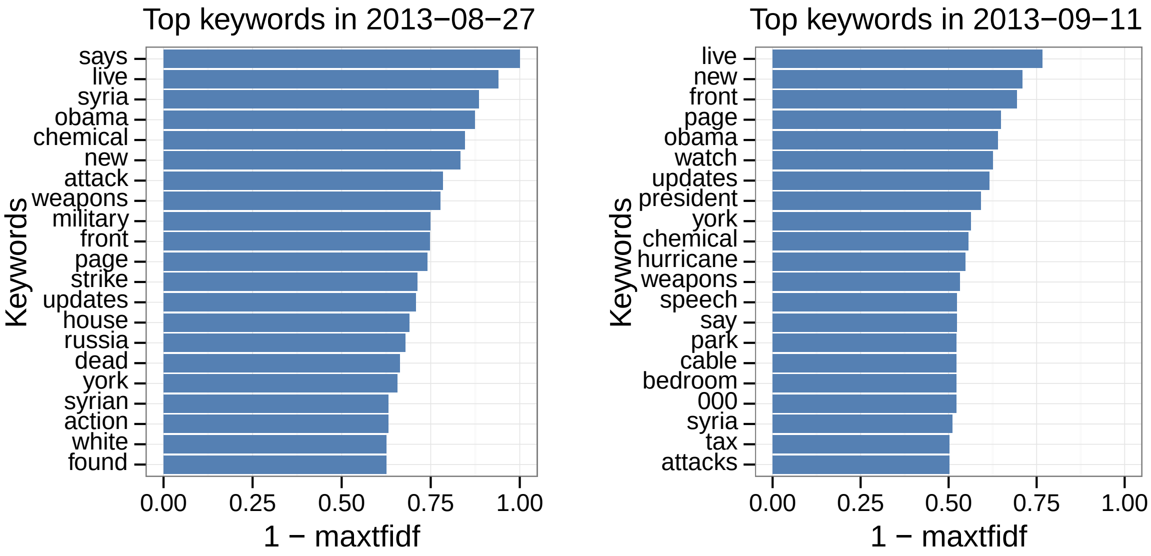

Figure 2: **Stopwords detection. Normalized $1-$ maxtf-idf score for data from August 27th (left) and August 28th (right) of 2013. The top score words for both plots are "says" and "live". We used the top score words to disconnect connected components of events.**

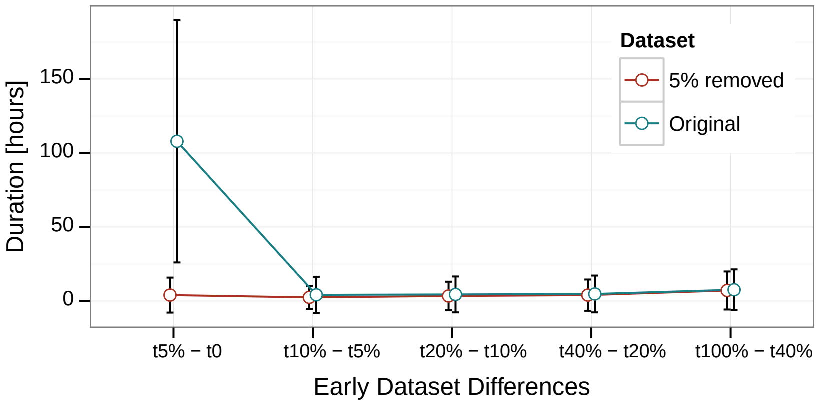

Figure 3: **Duration differences of events. The x-axis represents the categories of datasets: the first one (t5%-t0) represents the difference of time between the timestamp of the oldest tweet and the newest tweet in the first 5% of the tweets. The next one (t10%-t5%) corresponds to the difference between the newest tweet in the first 10% and the newest tweet in the first 5% of data, etc. After removing the first 5% of data, the time differences are roughly the same across all datasets.**

1.3 Validation of Data Collection

We performed experiments validating that merging keywords by forming connected components indeed produced meaningful groups of keywords representing an event. As a baseline, we used components obtained by merging random keyword pairs together. We evaluated how well a cluster is formed from the set of tweets obtained from connected components, comparing the cluster to the set of tweets obtained from random components. Connected components are expected to merge keyword pairs that belong to the same event, and hence would make better clusters when compared to merging random keyword pairs. The results are displayed in Figure 5. In this figure, each plot depicts a different metric that evaluates the quality of a cluster. These clustering metrics are summarized in Table 2. For better interpretation and visual clarity, in each of the plots, we sorted the clustering metrics obtained via connected components. We then rearranged the clustering metrics for the baseline according to the sorting order obtained from connected components. (This is the reason why the blue line is monotonically increasing.) This experiment was performed on one month of data (there are approximately 30 data points in each plot) between August 2013 and September 2013. We took all the keyword pairs obtained in a day and found the connected components as in Figure 4. For random components, we merged the keyword pairs randomly. We took precautions to make sure that the size of the connected components and random components per day were comparable. That is, if we had connected components of sizes 6, 6, and 5 formed from keyword pairs on particular day, we made sure that similarly sized random components were also formed from the keyword pairs of the same day. Also, to make sure that tweets from any one keyword pair do not dominate the tweet set, we sampled an equal number of tweets from each keyword pair, and the *same* sample of tweets is used to calculate the clustering metrics in both the connected components approach and the random components approach. The random baseline has been averaged over 3 different rounds of experimentation.

2 VQ Event Model

We introduce a novel vectorial representation based on a vector quantization of the interarrival time distribution, which we call "VQ-event model". The most representative interarrival times are learned from a large training corpus. Each of the learned interarrival times is called a *codeword*, and the entire set of the learned interarrival times, the *codebook*.

We represent an event e, belonging to a collection of events $\mathcal{E}$, as a tuple $(\mathcal{K}_e, \mathcal{M}_e)$, where $\mathcal{K}_e$ is a set of *keywords* and $\mathcal{M}_e$ is a set of *social media messages*. Both the keywords and the messages are related to a real-world occurrence. As explained in Section The keywords are extracted in order to succinctly describe the occurrence, and the messages are posts from users about the event.

To learn the most representative interarrival times we perform the following: for each $e \in \mathcal{E}$ with messages $\mathcal{M}_e = [m_1^e, m_2^e, \dots m_n^e]$ and their corresponding time-stamps $[t_1^e, t_2^e, \dots t_n^e]$ where $t_i \leq t_{i+i} \forall i \in [1, n]$, we compute all the interarrival times $d_i^e = t_i^e - t_{i-1}^e$ (the value of t_0 is

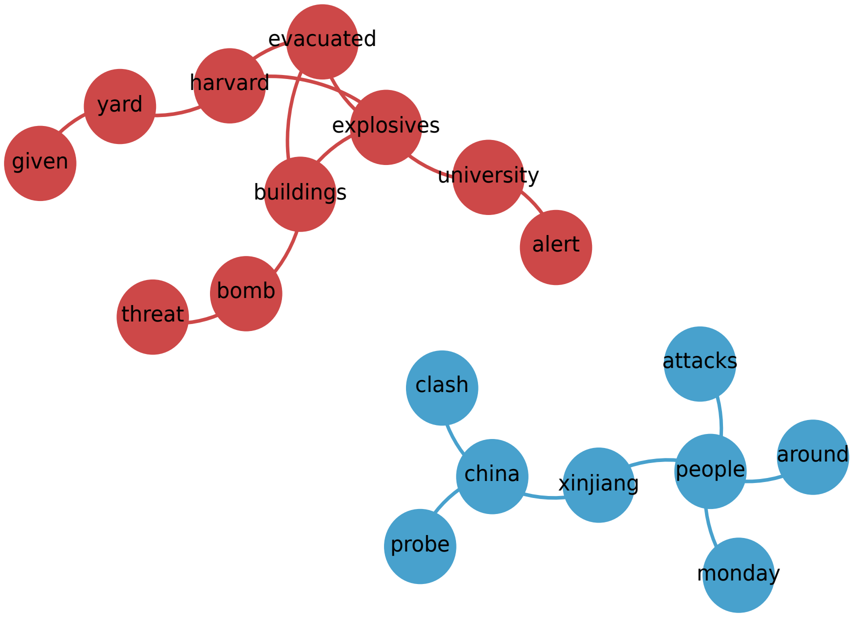

Figure 4: **This figure illustrates how we merge keyword pairs which represent the same event into larger components.**

Name	Metric	Meaning
I_1	$\sum_{i=1}^{k} \frac{1}{n_i} \sum_{(u,v)\in S_i} \text{sim}(u,v)$	Higher value is better
I_2	$\sum_{i=1}^{k} \sqrt{\sum_{(u,v)\in S_i} (u,v)}$	Higher value is better
E_1	$\sum_{i=1}^{k} n_i \frac{\sum_{v\in S_i, u\in S} \text{sim}(u,v)}{\sqrt{\sum_{(u,v)\in S_i} \text{sim}(u,v)}}$	Lower value is better
G_1	$\sum_{i=1}^{k} \frac{\sum_{v\in S_i, u\in S} \text{sim}(u,v)}{\sum_{(v,u)\in S} \text{sim}(v,u)}$	Lower value is better
G_1'	$\sum_{i=1}^{k} n_i^2 \frac{\sum_{v\in S_i, u\in S} \text{sim}(v,u)}{\sum_{(u,v)\in S_i} \text{sim}(u,v)}$	Lower value is better
H_1	$\frac{I_1}{E_1}$	Higher value is better
H_2	$\frac{I_2}{E_1}$	Higher value is better

Table 2: **This table lists the clustering metrics used in Figure 5.**

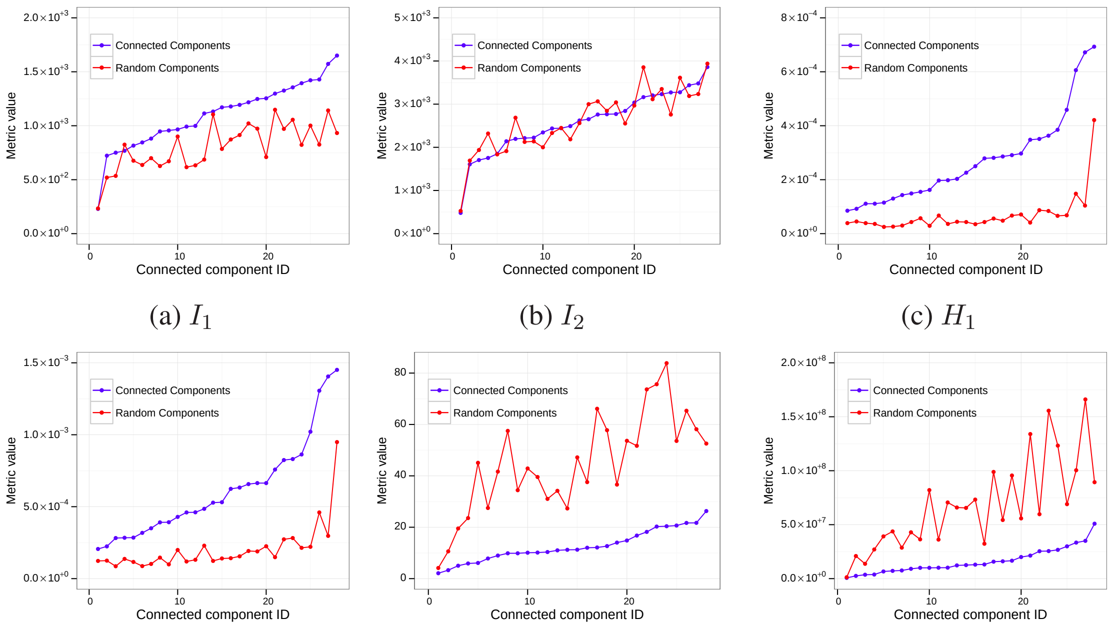

(a) I_1 (b) I_2 (c) H_1

(d) H_2 (e) G_1 (f) G'_1

Figure 5: **Each plot in this figure compares the quality of the cluster of tweets obtained from connected components and random components. The actual metric is shown in Table 2. In I_1, I_2, H_1, H_2 higher value is better. In G_1, G'_1, lower value is better. For visual clarity, the values obtained from connected components were sorted in ascending order, hence the blue line is monotonically increasing. The values obtained were rearranged in the same order as well.**

considered equal to t_1 for initialization purposes). Then, the values of d_i^e for all events in $\mathcal{E}$ are clustered to identify the *most representative* interarrival times.

Once the most representative interarrival times have been learned, the vector quantizations for each event is produced as follows: for each event, obtain all the interarrival times, and quantize each of the interarrival times to the closest codeword in the codebook. This process is summarized in Algorithm 3. Line 1 collects all of the interarrival times for all the events in $\mathcal{E}$ in **f**. Line 2 is a clustering algorithm which takes **f** and the number of clusters k as inputs and returns the centroids of the clusters as the output in **c**. The centroids can be thought of as the most representative interarrival times for the event set $\mathcal{E}$. After that, the interarrival times of each event e is vector quantized in terms of the centroids to obtain a k-dimensional real valued representation of the event (Line 4). In this representation, each entry is percentage of messages with that particular codeword as the interarrival time.

Algorithm 3 `learn_representation()`

Input: Event set $\mathcal{E}$, and number of codewords k in the codebook.
Output: A representation in $\mathbb{R}^k$ of each event $e = (\mathcal{K}_e, \mathcal{M}_e) \in \mathcal{E}$.
1: $\mathbf{f} \leftarrow \{d_i^e | m_i^e \in \mathcal{M}_e, e \in \mathcal{E}\}$
2: $\mathbf{c} \leftarrow \texttt{cluster}(\mathbf{f}, k)$
3: **for** $e \in \mathcal{E}$ **do**
4: $\quad \mathbf{e} \leftarrow \texttt{vq}(d_i^e, \mathbf{c})$
5: **end for**

3 High Activity Vs Low Activity Events

Once the events are converted into their VQ-event model representation, the goal is to identify groups of events such that all events belonging to a group have produced similar levels of activity in the social network. Events are considered to have produced similar levels of activity if the interarrival times between their social media posts are similarly distributed, implying a very much alike collective reaction from users to the events within the group. In order to identify groups of similar events, we perform clustering the event models. We sort the resulting groups of events from highest to lowest activity, according to the concentration of social media posts in the bins that correspond to short interarrival times. We consider the events that fall in the top cluster to be high-activity events as most of their interarrival times are concentrated in the smallest interval of the VQ-event model. Thus we end with four groups of events: high, medium-high, medium-low and low. shows a heatmap of the interarrival relative frequency for each cluster.

Through this section, we analyze different features for each of the event categories and compare them both qualitatively and quantitatively. We peformed two-tailed t-tests for a variety of features for events in the high-activity category, and compare it with the average values for the remaining events.

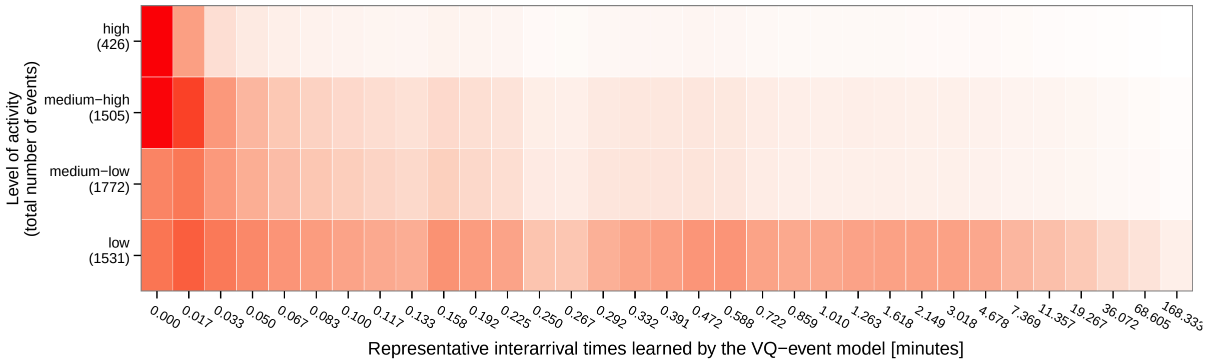

Figure 6: **The events were clustered to obtain four groups of events based on their interarrival times. Each row in the figure is the average representation of all events in that cluster. A darker cell represents a higher value.**

3.1 Information Forwarding Characteristics

We found that the high-activity events possess more information forwarding characteristics than other events. We present four features which support this argument. The features, their description and their values are listed in Table 3.

The `retweet_count` is generally higher for high-activity events. This feature is the fraction of retweets present in the event, log-normalized by the total amount of tweets in the event. A higher value suggests that people have a greater tendency to spread the occurrence of these events, and forward this information to their followers.

The `tweets_retweeted` is lower for high-activity events than for the rest. This feature is the number of tweets which have been retweeted, log-normalized by the total number of tweets in the event. This suggests that the high amount of retweets for the high-activity events actually originates from fewer tweets. This suggests that fewer tweets become popular and are retweeted several times.

The `retweets_most_retweeted` is the total number of retweets of the tweet that has been retweeted the most. This number is much higher for high-activity events than for low-activity events, suggesting that the most popular tweet indeed becomes very popular when the event is of high-impact.

3.2 Conversational Characteristics

We found that high-activity events in general tend to generate more conversation between users than the events in other categories. We observe this behavior through several features. Refer to Table 4.

The features `replies` and `norm_replies` both count the number of replies, but have been normalized slightly differently. Both have a higher value for high-activity events suggesting that high-activity events in general tend to spark more conversation between the users. The

Feature Name	Description	high-activity, others	Hypothesis, p-value
retweet_count	log(total retweet count in the event divided by total tweets in the event)	$2.205, 1.473$	$1, p = 0$
tweets_retweeted	log(number of tweets retweeted divided by total tweets in the event)	$-1.091, -0.964$	$1, p = 2.7 \times 10^{-5}$
retweets_most_retweeted	number of tweets of the most retweeted tweet	$284.491, 40.261$	$1, p = 0$

Table 3: **(Refer to Section 3.1.) This table lists all the features which characterize the information forwarding aspect of an event. In general, high-activity events tend to have higher values for information forwarding features than other events.**

Feature Name	Description	high-activity, others	Hypothesis, p-value
replies	log(total replies divided by total tweets)	$-1.4016, -1.6474$	$1, p = 10^{-4}$
norm_replies	log(number of replies divided by total number of unique users)	$-1.5796, -1.9294$	$1, p = 6.7 \times 10^{-4}$
tweets_replied	log(number of tweets which generated replies divided by total tweets)	$-1.7784, -2.0668$	$1, p = 0.001$
uniq_users_replied	log(unique users who have written a reply divided by total tweets)	$-1.7524, -2.0352$	$1, p = 0.001$

Table 4: **(Refer to Section 3.2.) This table lists all the features which characterize the conversational aspect of high-activity and remaining events. Using these features, we argue in Section 3.2 that high-activity events tend to invoke more conversation amongst users than their counterparts.**

Feature Name	Description	high-activity, others	Hypothesis, p-value
uniq_words	log(total unique words divided by total tweets)	$-0.1982, 0.1651$	$1, p = 0$
uniq_chars	log(total unique characters divided by total tweets)	$2.0009, 2.0456$	$1, p = 0$
uniq_hashtags	log(number of unique hashtags divided by total tweets)	$-1.1126, 0.8761$	$1, p = 0$
uniq_urls	log(number of unique urls divided by total tweets)	$-0.7194, -0.4951$	$1, p = 0$

Table 5: **(Refer to Section 3.3.) This table summarizes all the features that were used to study the topical focus characteristics of high-activity events.**

tweets_replied feature counts the number of tweets which have generated replies (it has been log-normalized by the total number of tweets in the event). This is also higher for high-activity indicating that such events on average have more tweets which invoke a reply from people. The uniq_users_replied feature counts the number of unique users who have participated in an conversation. Again, this number is found to be higher for high-activity events than for others suggesting that more users tend to engage in a conversation about these events. All these features collectively suggest that high-activity events tend to have a *conversational characteristic* associated with them.

3.3 Topical Focus Characteristics

We find that high-activity events have a lot more focus in terms of the topical content than the remaining events. This possibly suggests that when a news item is sensational, people seldom deviate from the topic of the news to other things.

We used four features listed in Table 5 to study the topic focus characteristics of high-impact events.

The number of unique words (uniq_words) and characters (uniq_chars) for high-activity events is lower than the remaining events suggesting that the information content for high-activity events is more focused than for the remaining events (as they do not need a diverse vocabulary). Hashtags on Twitter are a sequence of characters that follow the # symbol. Conventionally, their purpose is to indicate the topic of the tweet. Again, this number (uniq_hashtags; log-normalized by the total number of tweets) is lower for the high-activity events than for the remaining events. The number of unique URLs (uniq_urls; which can be taken to interpret similar semantics as the hashtags) is also lower for high-activity events than for the rest.

3.4 Early prediction of high-activity events

The results from sections 3.1, 3.2 and 3.3 suggest that high-activity events differ considerably from other events in terms of how they are received by the users and in terms of the response

	Early 5% Tweets		All Tweets	
	high-activity	others	high-activity	others
high-impact	194	232	230	196
non-high-impact	43	4 765	47	4 761

Table 6: **Confusion matrix while predicting the top 8% of events as high-activity. The predictions were made using the early 5% of the tweets, and by using all the tweets from the event.**

	Early 5% Tweets				All Tweets			
	FP-Rate	Precision	Recall	ROC-area	FP-Rate	Precision	Recall	ROC-area
high-activity	0.009	0.819	0.455	0.900	0.01	0.830	0.540	0.945
others	0.545	0.954	0.991	0.900	0.460	0.960	0.990	0.945

Table 7: **Classification results of detecting whether an event from the top 8% is high-impact or not while predicting from features extracted from the earliest 5% of the tweets and from all the tweets belonging to the event.**

they invoke from the network.

In the next phase, our goal is to supervised machine learning only the early tweets of an event to predict whether an event will generate high-activity or not. A list of all the features used for classification is shown in Table 8. The classification was carried using logistic regression provided by the Weka package. The data was split approximately into $60 - 20 - 20$ of training, test and validation sets and the results were averaged over 5 runs of experiments.

Table 7 illustrates the prediction results from the earliest 5% of the tweets tweets, and from using all the tweets. We the false positive rate using only the early tweets is almost as good as the false positive rate using all the tweets. The same observation holds for the metrics precision and ROC-area as well. However, we observe an 18% increase in the recall (0.455 to 0.540). This suggests that some high-activity events perhaps do not start displaying their unique characteristics well enough in their early stages.

**Feature Name**	**Normalized By**	**Normalization Method**
`component_size`	None	
`total_seconds`	`total_tweets`	$\log(x) - \log(y)$
`total_tweets`	None	
`total_retweets`	`total_tweets`	$\log(x) - \log(y)$
`total_tweets_retweeted`	`total_tweets`	$\log(x) - \log(y)$
`retweets_most_retweeted`	`total_retweets`	$\log(x) - \log(y)$
`total_mentions`	`total_tweets`	$\log(x) - \log(y)$
`total_unique_mentions`	`total_mentions`	$\log(x) - \log(y)$
`total_tweets_with_mention`	`total_tweets`	$\log(x) - \log(y)$

Continued on next page

Table 8 – *Continued from previous page*

Feature Name	Normalized By	Normalization Method
total_tweets_with_mostfrequent_mention	total_tweets_with_mention	$\log(x) - \log(y)$
total_hashtags	total_tweets	$\log(x) - \log(y)$
total_unique_hashtags	total_hashtags	$\log(x) - \log(y)$
total_tweets_with_hashtag	total_tweets	$\log(x) - \log(y)$
total_tweets_with_mostfrequent_hashtag	total_tweets_with_hashtag	$\log(x) - \log(y)$
total_urls	total_tweets	$\log(x) - \log(y)$
total_unique_urls	total_urls	$\log(x) - \log(y)$
total_tweets_with_url	total_tweets	$\log(x) - \log(y)$
total_tweets_with_mostfrequent_url	total_tweets_with_url	$\log(x) - \log(y)$
total_unique_verified_users	total_verified_users	$\log(x) - \log(y)$
total_verified_users	total_tweets	$\log(x) - \log(y)$
total_unique_users	total_tweets	$\log(x) - \log(y)$
total_replies	total_unique_users	$\log(x) - \log(y)$
total_tweets_first_replied	total_tweets	$\log(x) - \log(y)$
total_unique_users_replied	total_unique_users	$\log(x) - \log(y)$
total_tweets_replied	total_tweets	$\log(x) - \log(y)$
total_words	total_tweets	$\log(x) - \log(y)$
total_unique_words	total_words	$\log(x) - \log(y)$
total_characters	total_tweets	$\log(x) - \log(y)$
total_rt_count	total_tweets	$\log(x) - \log(y)$
total_fav_count	total_tweets	$\log(x) - \log(y)$
total_positive_sentiment	total_tweets	x/y
total_negative_sentiment	total_tweets	x/y

Table 8: **List of features used for characterization and classification. The "Normalization Method" column corresponds to the method used to normalize the value of the first column using the value of the second column. For example, the total number of retweets was normalized dividing it by the total number of tweets, and then taking the logarithm. Zero values were replaced by 10^{-8}.**

References and Notes

1. Karen Sprck Jones. A statistical interpretation of term specificity and its application in retrieval. *Journal of Documentation*, 28:11–21, 1972.

2. Pang-Ning Tan, Michael Steinbach, and Vipin Kumar. *Introduction to Data Mining, (First Edition)*. Addison-Wesley Longman Publishing Co., Inc., Boston, MA, USA, 2005.
